# Acoustofluidic Actuation of Living Cells

**DOI:** 10.3390/mi15040466

**Published:** 2024-03-29

**Authors:** Yue Wu, Junyang Gai, Yuwen Zhao, Yi Liu, Yaling Liu

**Affiliations:** 1 Department of Bioengineering, Lehigh University, Bethlehem, PA 18015, USA; yuwc19@lehigh.edu; 2 Department of Mechanical and Aerospace Engineering, Monash University, Clayton, VIC 3800, Australia; junyang.gai@gmail.com; 3 Department of Mechanical Engineering and Mechanics, Lehigh University, Bethlehem, PA 18015, USA; yuz721@lehigh.edu; 4 School of Engineering, Dali University, Dali 671000, China

**Keywords:** acoustofluidics, acoustofluidic manipulation, acoustofluidic sorting, acoustofluidic patterning, acoustofluidic printing, acoustofluidic delivery

## Abstract

Acoutofluidics is an increasingly developing and maturing technical discipline. With the advantages of being label-free, non-contact, bio-friendly, high-resolution, and remote-controllable, it is very suitable for the operation of living cells. After decades of fundamental laboratory research, its technical principles have become increasingly clear, and its manufacturing technology has gradually become popularized. Presently, various imaginative applications continue to emerge and are constantly being improved. Here, we introduce the development of acoustofluidic actuation technology from the perspective of related manipulation applications on living cells. Among them, we focus on the main development directions such as acoustofluidic sorting, acoustofluidic tissue engineering, acoustofluidic microscopy, and acoustofluidic biophysical therapy. This review aims to provide a concise summary of the current state of research and bridge past developments with future directions, offering researchers a comprehensive overview and sparking innovation in the field.

## 1. Introduction

Acoustofluidics, an emerging field blending acoustics and fluid mechanics, has garnered widespread interest for its applications in fundamental research and biomedicine [1,2,3]. At its core, the manipulation of living cells with acoustofluidics leverages the differential acoustic radiation and fluid drag forces acting on objects with distinct physical and mechanical properties, resulting in unique motile behaviors within an acoustofluidic environment. Significantly, acoustofluidic methods circumvent the need for additional modifications that could perturb biological activity, such as magnetic binding or immunoassay procedures.

In contrast to optical tweezers, which utilize lasers and risk thermal damage to biological samples, acoustofluidics employs mechanical wave propagation within fluids, presenting no such risk of thermal injury. Furthermore, the acoustic field can be finely tuned by altering the frequency and voltage of the input signal, affording precise remote control. The corresponding acoustic wavelengths in acoustofluidic setups span from several micrometers to hundreds of micrometers, conveniently matching the scale of single cells and micro-tissues [4]. This positions acoustofluidics as an ideal technology for contactless manipulation of living cells across a spectrum of sizes, from micrometers to millimeters, in biomedical analysis.

In view of the vigorous development of the acoustofluidic field, many previous review articles have been published to present in-depth descriptions of the basic principles [5,6,7,8]. This review aims to provide a concise summary of the latest applications and current evolution of acoustofluidic technology in the manipulation of living cells, ranging from suspended single red blood cells (RBCs) to zebrafish larvae. We spotlight and discuss acoustofluidic sorting, patterning for tissue engineering, microscopy, biophysical therapy, and other innovations within the field. Our aim is to illuminate the characteristics and advancements of acoustofluidic technology, fostering an understanding that may spur novel ideas among researchers, thereby catalyzing the interpretation of this technology from the lab to clinical applications. It is envisioned that acoustofluidics will become integral to both foundational research in laboratories and practical clinical applications in hospitals in the foreseeable future.

## 2. Suspended Single Cells in the Acoustofluidic Field

Acoustic waves are typically generated by piezoelectric transducers. These transducers expand or contract in response to an applied voltage, converting electrical energy into mechanical vibrations that produce the propagation of acoustic waves in the surrounding medium. The acoustic mechanism of bulk acoustic wave (BAW) devices is similar to that of surface acoustic wave (SAW) devices. Due to limitations of material and manufacturing processes, the upper limit of a BAW device’s transducer frequency probably does not exceed a few megahertz. SAW devices can be manufactured by sophisticated semiconductor processes, and the upper frequency limit can reach hundreds of megabytes or even GHz. Here, we take the generation process of surface acoustic waves as an example to illustrate the forces exerted on microscopic particles in the acoustic flow field.

Surface acoustic waves (SAWs), produced by applying an oscillating electrical signal on interdigital transducers (IDT) on piezoelectric material, are acoustic waves that propagate along a surface with a majority of the acoustic energy confined in the vicinity of the surface [9]. Primarily, SAWs have been utilized for telecommunication applications such as signal filtering for radio-frequency (RF) devices, due to the convenience of fabricating SAW chips by producing IDT patterns with achievable dimensions using standard photolithography on a substrate of reasonable size [10]. The anisotropy of a piezoelectric material dictates the waveforms of the generated SAWs. Rayleigh waves, consisting of a longitudinal and transverse motion confined within two wavelengths in depth, are the most common SAW waves for SAW-based microfluidic applications [11]. Other SAW modes, such as shear horizontal waves which involve a horizontal motion and a longitudinal motion, are less applied in microfluidic applications and are not discussed in this review. Surface acoustic waves that are generated by a single set of IDTs are known as travelling surface acoustic waves (TSAWs), while two TSAWs propagating against each other will result in a one-dimensional standing surface acoustic wave (SSAW) field, with pressure nodes and pressure antinodes appearing at fixed locations. When a TSAW impinges on a fluid, part of the TSAW refracts into the fluid, in the form of a longitudinal wave, known as a leaky wave. When the leaky wave propagates inside the viscous fluid, due to the dissipation in the fluid, a time-averaged body force is generated, acting on the fluid along its propagation direction, in turn causing fluid vibrations. This fluid motion is rotational, conforming to the no-slip boundary condition of the wall. This non-linear, steady-state flow phenomenon caused by sound waves interacting with fluid is known as acoustic streaming.

For a particle immersed in an acoustic streaming field with an initial velocity *u*, it is subjected to a flow drag force induced by the steady-state streaming velocity field, given by the Stokes drag formula [12,13]:

$$F_{D}=-6\mu_{f}\pi R_{p}v$$

*μ_f_* depicts fluid viscosity, *R_p_* portrays cell radius, and *v* is the relative velocity between the cell and the liquid fluid flow. This force counterbalances the ARF, enabling precise cell positioning. The acoustic field can usually be constructed by the interdigital transducers (IDT) on the piezoelectric wafer [14] or lead zirconate titanate (PZT) transducer [15]. These fields can be categorized into two types: those combined with continuous fluid flow, primarily used for cell sorting, and those combined with discontinuous fluid flow, typically employed in tissue engineering [16]. 

When an object is suspended in an acoustic pressure field in a fluid, it is subjected to a time-average force caused by the vibrating fluid volume at high frequencies (kHz or MHz), which is known as the acoustic radiation force (ARF). There are three different scenarios that give rise to the resultant force, namely the interaction with the incident sound wave and the particle, the scattered sound wave on the particle, and finally the sound wave within the particle due to transmittance. The resultant pressure field is dependent on the physical properties of the objects including density, size, and shapes, with two contributing components. The first one is primary ARF, which results from the direct interaction between incident waves and the objects suspended in the fluid, and the second one is secondary ARF, also known as the Bjerknes force, caused by the scattered acoustic waves from other suspended matter acting on the objects. 

Due to the nature of cells, the ARF *F_R_* is usually defined in cases where compressible spherical particles are suspended in an inviscid fluid [17,18,19]:

$$F_{R}=-(\frac{\pi p_{0}^{2}V_{p}\beta_{f}}{2\lambda })\emptyset \left(\beta ,\rho \right)\sin\left(\frac{4\pi x}{\lambda }\right)$$

$$\phi \left(\beta ,\rho \right)=\frac{5\rho_{p}-\rho_{f}}{2\rho_{p}+\rho_{f}}-\frac{\beta_{p}}{\beta_{f}}$$

where ϕ is the acoustic contrast factor, representing the difference in compressibility (*β*) and density (*ρ*) between the cells (*p*) and the fluid (*f*). Here, *ρ*_0_ and *λ* denote the acoustic pressure and wavelength, respectively, and *x* is the spacing to the nearest pressure node. Cells’ contrast factor is defined as positive and they are propelled towards the pressure nodes, facilitating manipulation [20]. 

Given by its characteristics, ARF has been utilized extensively for patterning, sorting, and manipulating targeted objects in lab-on-chip applications. The flow pattern of acoustic streaming, correlated with confined fluid shape, the relative position of refracted SAWs in fluid, as well as the operating frequency, could be utilized for biomedical applications including fluid mixing and drug delivery. 

In some scenarios, when acoustic waves propagate in a fluid medium, it causes the fluid to undergo stable flow motion, that is, acoustic streaming [21,22,23,24,25]. In the acoustofluidic field, the forces acting on particles’ movement mainly come from the interaction between acoustic waves and the streaming. These forces can be decomposed into two main components: the primary acoustic radiation force [26] and streaming-induced viscous drag force [27]. The dominance of the acoustic radiation forces or streaming-induced drag force on particles in an acoustofluidic field depends on a variety of factors, including the properties of the acoustic field and the properties of the particles and surrounding fluid [28,29,30]. Generally speaking, under certain conditions, such as high acoustic pressure amplitude, large particle size, and low-viscosity fluids, the primary acoustic radiation force dominates the particle motion. In the case of low acoustic pressure amplitude, small particle size, and high-viscosity fluids, streaming drag force dominates. Nonetheless, there are some side effects under extreme conditions, such as overheating, shear force from acoustics-induced streaming, and cavitation, which can directly damage cell structures [31,32]. Most acoustofluidic devices are used only to guide cells’ spatial translocation and are essentially biocompatible [33].

## 3. Acoustofluidic Cell Sorting

Acoustofluidic cell sorting capitalizes on cells’ physical and mechanical properties to dictate their movement within a field [34,35,36]. A prime example of this acoustofluidic technology’s application is the sorting of living cells from raw blood samples, offering a less invasive alternative to conventional methods like immune antibody capture, which may alter cell viability and biochemical properties [37,38,39,40,41,42,43,44]. Although immunomagnetic bead capture is currently still the gold standard for clinical cell sorting [45,46,47], this acoustic sorting technology enables the sorting of blood cells while preserving their inherent characteristics, allowing for further downstream expansion or biochemical characterization [33,48]. The process of acoustofluidic cell sorting can be likened to the motion of objects in a flat projectile trajectory, combining horizontal linear motion with vertical free-fall. In this analogy, the propagation direction of the acoustic field is usually perpendicular or at an acute angle to the flow direction of the fluid field [49], creating a transverse force component that deflects the cells from the flow direction.

The trajectory of the cells is influenced by the acoustic field intensity and the exposure duration of them to the acoustic field, determining their eventual entry into distinct collection channels for sorting purposes. For illustrative purposes, we discuss the application of acoustofluidic sorting of living blood cells in two categories: automated high-throughput sorting and selective high-precision sorting.

### 3.1. Automatic High-Throughput Sorting

Blood contains a high concentration of cells of varying sizes, such as plasma, platelets (PLTs), white blood cells (WBCs), and red blood cells (RBCs), each with distinct dimensions [50]. For example, the main components of blood are RBCs with a diameter of approximately 6 μm, white blood cells (WBCs) with a diameter of approximately 13 μm, platelets (PLTs) with a diameter of approximately 3 μm, and plasma. 

These different types of blood cells each have significant concentrations [51,52]. These size differences result in varied force impacts in the acoustofluidic field, leading to different cell trajectories. Moreover, quantifying various blood cell concentrations is often essential for clinical diagnoses [53,54,55]. Thus, acoustofluidic cell sorting emerges as an optimal solution for high-throughput separation of the blood components [42]. 

**Figure 1:**
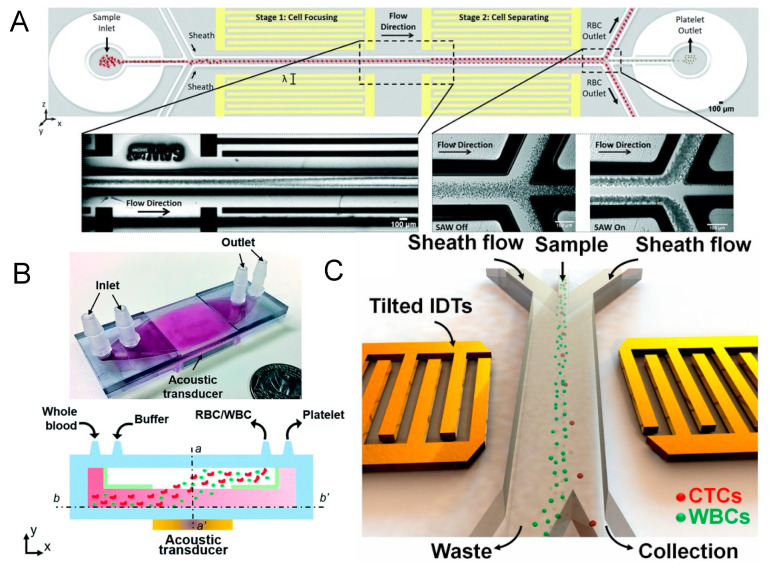
(**A**) Schematic illustration and images of the standing surface acoustic wave (SSAW)-based device for PLT separation. Reprinted with permission from [56]. (**B**) Photograph of the acoustic platelet separation device and schematic illustration of the acoustofluidic PLT separation process. Reprinted with permission from [62]. (**C**) Schematic illustration of the tilted-angle SSAW device for circulating cancer cell separation. Reprinted with permission from [63].

Richard et al. introduced an acoustofluidic device capable of separating platelets from RBCs in whole blood with high throughput. This device operates with a standard standing surface acoustic wave (SSAW)-driven resonator [56]. By establishing a stable standing acoustic field over a sufficient area, the cells in flow are directed either perpendicularly or obliquely to the flow, leading to their segregation into different channels (Figure 1A). Here, two perpendicular standing acoustic fields are constructed in relation to the hydrodynamic flow. First, the RBCs and PLTs are focused into a single profile by two sheath flows. Fine-tuning the SSAW frequency and phase, along with the microfluidic channel dimensions and positioning, results in a targeted separation of RBCs and PLTs, facilitating downstream analysis. Specifically, the first SSAW field (13 MHz, 150 mW) forms an acoustic pressure distribution in the first half of the channel with a pressure node sandwiched between two pressure antinodes, while the second SSAW field (12.7 MHz, 120 mW) forms an acoustic pressure distribution with two pressure nodes sandwiching one pressure antinode in the second half of the channel. As a result, the first SSAW field enhances cell stream narrowing in the fluidic channel center. Then, RBCs, which are significantly larger than PLTs, migrate to the two pressure nodes close to both sides of the channel when passing through the second SSAW field. The PLTs remain at the antinode in the middle of the channel while flowing straight forward with the flow. In this way, the PLTs are separated from the RBCs for potential downstream analysis.

Similar proof-of-concept studies are abound [57,58,59,60]. However, these SAW-based sorting devices remain precision instruments with high associated costs, requiring micro–nano-manufacturing in cleanrooms. Additionally, the effective acoustic field space is limited, indicating potential for throughput enhancement. Gu et al. innovatively utilized commercially available low-frequency piezoelectric transducers (610 KHz) with high-throughput manufacturable plastic channels (Figure 1B), successfully creating a scalable BAW acoustofluidic platelet sorting device with broad production potential [61,62]. Although ultracentrifugation remains the clinical gold standard for cell sorting, the low-cost, user-friendly, and functional acoustofluidic sorting equipment presented by Gu betokens a significant step towards practical point-of-care testing applications.

### 3.2. Selective High-Precision Sorting

Selective high-precision acoustofluidic sorting is particularly valuable for isolating rare cellular targets from blood components, such as isolated circulating tumor cells (CTCs) from patient blood samples (Figure 1C) [63]. These cells typically occur at a low frequency, approximately 10 CTCs per mL of whole blood [64,65,66]. To enhance sorting accuracy, researchers often dilute the sample to align cells in a single-file flow, thereby maintaining a uniform intercellular distance and allowing cells to pass through the detection and sorting zones sequentially. This necessitates an acoustofluidic device with a sufficiently narrow acoustic coverage zone, designed to target individual cells without affecting adjacent non-target cells [67]. 

**Figure 2:**
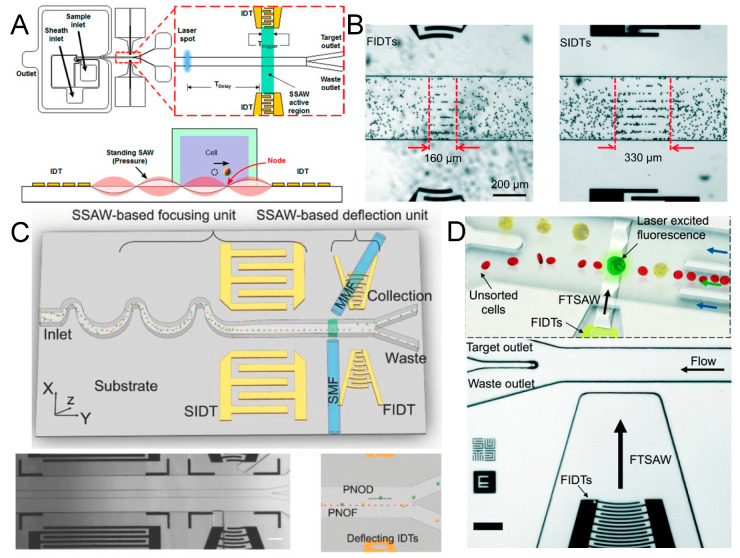
(**A**) Schematic illustration of a standing surface acoustic wave (SSAW)-based acoustofluidic single-cell sorting device indicating the focusing region and channel cross section delineating the sorting mechanism. Reprinted with permission from [68]. (**B**) Images of SSAW-based particle patterning by FIDTs (left) and SIDTs (right). Reprinted with permission from [70]. (**C**) Schematic illustration of the SSAW-based FACS chip and microscopic image of the focusing and sorting units. Reprinted with permission from [71]. (**D**) Schematic illustration and micrograph of the fluorescence-activated sorting process in focused traveling surface acoustic wave-based sorting chip. Reprinted with permission from [78].

A significant advancement was reported by Nawaz et al. in 2016 with a fluorescence-activated acoustofluidic cell sorter, utilizing a standard interdigitated transducer (SIDT) pair to create a SSAW working zone (−2.8 dBm, 33.8 MHz) (Figure 2A). The device featured an IDT with a short active region of 120 μm. Upon fluorescence-based detection of target cells, the system triggers the sorting module after a pre-defined delay, ensuring that the sorting action coincides with the target cell’s presence within the effective area. This synchronization enables the integration of identification and sorting with single-cell accuracy [68]. 

Subsequent research further indicated that focused IDT (FIDT) configurations can yield smaller areas of impact (Figure 2B), allowing for closer spacing of cells and higher throughput efficiency [69,70]. In 2018, Ren et al. reported an FIDT-based fluorescence-activated cell sorter (32.8 MHz) achieving a throughput of 2500 events per second (Figure 2C) [71]. Moreover, sorters based on traveling waves generated by a single FIDT are gaining popularity [72,73,74,75,76,77]. Although the acoustic potential energy distribution in a traveling acoustic wave field may not be as uniform and stable as in a standing acoustic wave field, the presence of a single target within the action area, coupled with a transient high-energy traveling wave, is sufficient to propel the target cell a considerable longitudinal distance.

Ma et al. introduced a system utilizing a focused traveling surface acoustic beam to deflect targets for fluorescence-activated cell sorting (FACS) in 2017 [78]. The system is reportedly capable of sorting targeted single cells at kHz rates, while preserving post-sorting cell viability above 95% (Figure 2D). Building on this, Nawaz et al. recently integrated image-based cell analysis with the focused traveling surface acoustic wave-based cell sorting system [79]. These innovations suggest that acoustofluidic sorters are poised for future integration with real-time analysis systems, offering enhanced functionality and efficiency [80,81]. 

## 4. Acoustofluidic Patterning of Living Cells for Tissue Engineering

**Figure 3:**
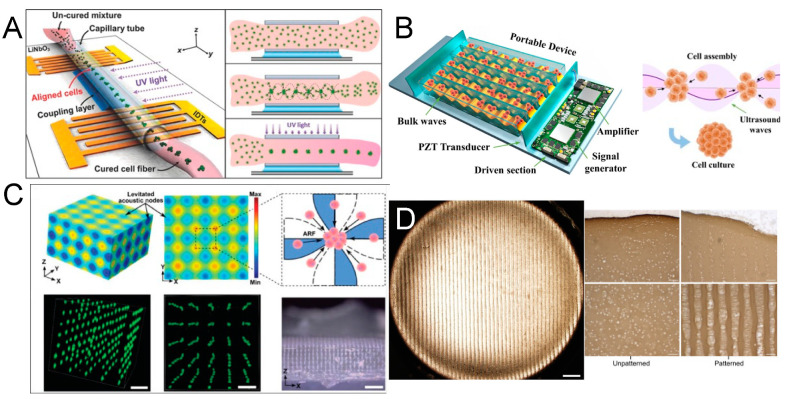
(**A**) Schematic illustration of the entire process for standing surface acoustic wave (SSAW)-based fiber generation with the patterned cell inside. The cells are resuspended in the light-curable hydrogel solution and loaded into a capillary tube. Then, the SSAW is activated to align the cells in the polymerized hydrogel fiber. Reprinted with permission from [87]. (**B**) Schematic diagram of the cell assembly in a portable acoustofluidic device. Reprinted with permission from [105]. (**C**) Acoustic pressure field generated by a 3D acoustic assembly device, and suspended cells are periodically distributed in a 3D dot-matrix pattern. Reprinted with permission from [106]. (**D**) Acoustic cell patterning can be used to produce hyaline cartilage tissue models, maintaining deep zone cytoarchitecture. Reprinted with permission from [111].

Acoustofluidic patterning, which capitalizes on stable standing wave fields in a discontinuous fluid environment, is gaining traction in regenerative medicine and biomedicine [82,83]. This technique can be likened to a microscale application of Faraday’s classic Chladni plate experiment, exploiting the non-contact, remote manipulation capabilities of acoustofluidics to precisely position live cells without compromising their viability, thus making it exceptionally suitable for tissue engineering endeavors [84,85]. Based on the non-contact, remote manipulation and biocompatible characteristics of acoustofluidics on live cells, a stable standing wave field can relocate to a specific location without affecting the cell state, which makes it very suitable for tissue engineering-related projects [86]. Particularly, in applications where acoustofluidic patterning is combined with light-curable hydrogels, the acoustic field initially redistributes cells within the pre-gel solution. Upon gel solidification, the cells’ spatial arrangement is fixed, maintaining the pattern even after the acoustic influence is removed (Figure 3A) [87,88]. A typical approach involves creating one- or two-dimensional standing wave fields, with the former arranging cells into parallel line arrays and the latter into dot-matrix patterns [89,90]. Here, the randomly distributed cells are redesignated to the nearest pressure nodes, generally spaced at half-wavelength intervals, with the node’s size—and hence the number of cells it can gather—proportional to the wavelength and the original cell suspension concentration [91,92]. The selection of the wave field orientation and wavelength is regulated by the unique requirements of the specific application. Acoustofluidic patterning is commonly employed in the high-throughput production of cell spheroids, offering a more efficient and controlled alternative to traditional methods that rely on passive cell aggregation in well plates [93,94,95,96,97,98,99,100,101,102,103]. This is often time-consuming and lacks control over spheroid uniformity. This active acoustofluidic patterning assembly process ensures uniform spheroid size by confining a similar number of cells at each pressure node, thus enhancing uniformity and reducing formation time [104]. 

Luo et al. introduced an integrated BAW acoustic chip for generating cell spheroids, using a pair of flat identical piezoelectric transducers aligned parallel at both ends of capillary tubes (Figure 3B). When activated by a low-power signal (0.1 W), a series of stable one-dimensional standing wave fields (from 230 kHz to 1.1 MHz) form, causing cells to coalesce at pressure nodes acting as invisible micro-wells to confine the cells. After a brief incubation, uniform cell spheroids are produced, primed for drug testing [105]. Notably, the group replaced bulky power amplifiers and signal generators with compact circuitry, making the device portable and accessible for mass production and use in biological labs without specialized piezoelectric expertise. Extending this concept, Miao et al. developed a vertical, multi-layered approach for BAW acoustofluidic cell cluster patterning, effectively creating a three-dimensional cell lattice and, thus, expanding the method’s theoretical capacity (Figure 3C). If integrated with Luo’s platform, this innovation could herald a new era of high-throughput, low-cost, automated spheroid generation equipment [106]. In parallel with the trajectory of 3D bioprinting, the prospect of commercially available acoustofluidic devices promises to significantly advance the field of biomedical engineering.

In applications where parallel line arrays are desired, such as in the engineering of strip-structured tissues like blood vessels, acoustofluidic patterning has demonstrated significant potential [107,108,109]. Kang et al. aligned endothelial cells into parallel arrays using acoustofluidics, which improved endothelial cell–cell interactions and gene expression, thus enhancing vascular tissue regeneration [110]. Similarly, Armstrong et al. employed ultrasound standing waves (6.7 MHz) to arrange living chondrocytes into anisotropic arrays with high resolution, showing improved articular cartilage regeneration (Figure 3D) [111]. While the patterning achievable with acoustofluidics may not offer the flexibility of 3D printing, its single-cell-level precision opens up possibilities for the regeneration of specialized and structurally specific tissues [112,113,114]. It is worth talking about acoustofluidic hologram patterning technology, which can shape sound waves into desired complex patterns [115,116,117]. Unlike traditional traveling wave interference, the mechanism of acoustofluidic holography is that when a planar acoustic wave strikes a 3D-printed mask template with the desired front phase encoded in the surface profile, it is affected by a bump pattern on the template. Its phase and amplitude are modulated according to a predetermined algorithm, thereby forming a complex-shaped pressure distribution. In this way, the spatial information of the tangible patterns on the template mask is transmitted to space through invisible acoustic waves [118]. Melde et al. even presented a 3D holographic assembly of cells and microgel beads in liquid in a centrifuge tube, and this is encouraging for tissue engineering and additive manufacturing fields [119,120,121,122,123]. Despite its great promise, to date, there have been few reported applications of this technology in the manipulation of living cells. One of the main limitations may come from the manufacturing of acoustofluidic hologram templates [124]. The range of materials compatible with acoustic properties is limited, and so are the available processing techniques, resulting in limited resolution. As exploration continues to advance, this technology promises to be truly revolutionary [125,126].

## 5. Acoustofluidic Microscopy

**Figure 4:**
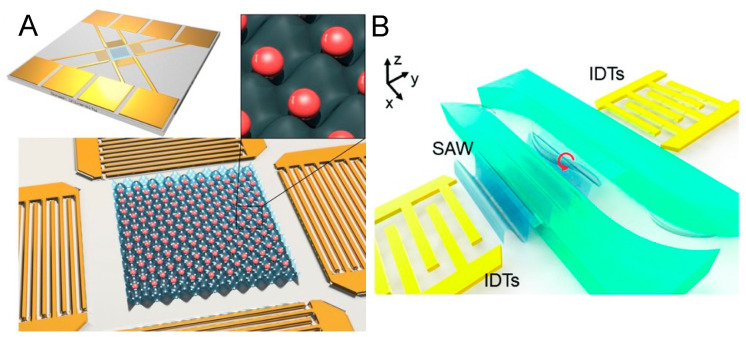
(**A**) Schematic illustration of standing surface acoustic wave (SSAW)-based one cell per acoustic well (OCPW) device. Two columns of standing waves perpendicular to the propagation direction are superimposed on each other to form an array of potential energy wells. Reprinted with permission from [129]. (**B**) Schematic illustration of a surface acoustic wave (SAW)-based acoustofluidic chip, which is capable of on-demand rotation of *C. elegans* for microscopic imaging. The red arrow stands for the rotation direction of acoustic streaming vortex. Reprinted with permission from [134].

Another important topic that has gained interest recently is the ability to manipulate living organisms for microscopic examination; such tasks can be challenging when dealing with living organisms [127]. Precisely manipulating the physical position of living objects is of great significance for multiple biomedical research applications such as investigating single-cell analysis and imaging motile organism morphology [128]. In 2015, Collins et al. reported a two-dimensional SSAW field with an internodal spacing of single-cell level [129]. The group reported that the minimum wavelength of acoustic waves was 15 μm and demonstrated the acoustofluidic single-cell patterning array (201 MH) of individual RBCs (Figure 4A). This project hints at the technology’s potential for regular and controllable distribution of single cells for microscopic observation. Recently, Yang et al. announced an acoustofluidic tweezer platform for single-cell profiling at high throughput [130]. They loaded a cell suspension into a time-effective Fourier-synthesized harmonic acoustic field. The acoustic patterning could efficiently distribute single cells into lattice grids to avoid cell overlapping in the microscopic imaging region. Compared with conventional flow cytometry, cells lined in a row flashing past in front of a camera, this acoustofluidic method can maintain the spatial distribution of individual cells under a microscope for a long time, which is undoubtedly more conducive to multi-angle, high-precision, and long-term microscopic analysis. Furthermore, they even demonstrated that acoustic tweezers could simultaneously pair and separate more than 100 pairs of cells in suspension, enabling the quantification of cell-to-cell interaction within these pairs. This suggests that this technology may be used in the future for high-throughput killing capability screening and retrospection of immune cells, which may contribute to the development of cell therapies, such as the development of CAR-T cells for antitumor treatment [131,132]. 

In addition to single-cell analysis, acoustofluidics also contributes in living microorganism microscopy [133]. *Caenorhabditis elegans* (*C. elegans*) has long been a classic model organism for developmental biology research. Controllable rotational manipulation of *C. elegans* is essential in three-dimensionally interrogating organism morphologies, tissue structure, and organs at desired orientations. Zhang et al. used a SAW (19.32 MHz) to generate a streaming vortex distribution inside a microfluidic channel (Figure 4B) [134], thereby achieving bidirectional rotation of elegans for high-resolution microscopy from different orientations [135]. The technology’s potential does not stop there, though. The zebrafish is another classic and important model animal. It has higher tissue complexity, a larger body size, and a more complex spatial distribution of organs among vertebrae. As a subject of preclinical studies of different drugs, a comprehensive phenotypic evaluation of zebrafish is necessary. Chen et al. presented a SAW-based acoustofluidic rotational tweezing platform to generate vortex acoustic streaming for zebrafish larvae manipulation [136]. The reported acoustofluidic system enables non-contact rapid rotation of the zebrafish body, accomplishing multispectral imaging of their internal organs from different viewing angles. It is worth emphasizing that all these acoustofluidic microscopy systems have good biocompatibility and are friendly to the living objects being characterized.

## 6. Acoustofluidic Biophysical Therapy

**Figure 5:**
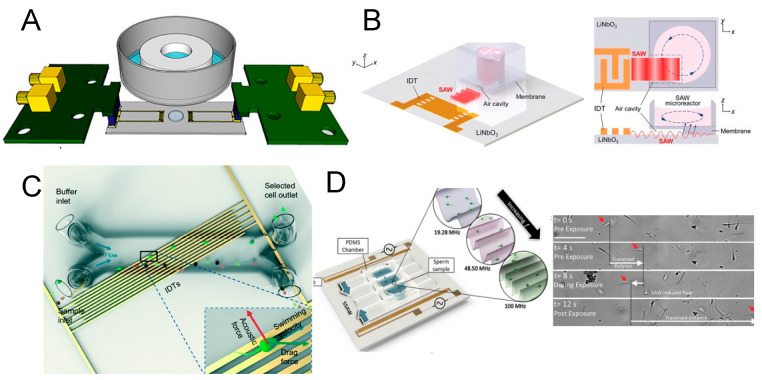
(**A**) Schematic illustration of a surface acoustic wave (SAW)-driven device for dynamic cell cultures. Acoustic streaming is activated with negligible heating in a dish to promote cell proliferation. Reprinted with permission from [138]. (**B**) Schematic illustration of a SAW-based microreactor system for dynamic immune cell culture. Acoustic streaming is triggered to adjust cytotoxic activity of natural killer cells. Reprinted with permission from [140]. (**C**) Schematic illustration of a SAW-based microfluidic sperm selection device. The sperms with better motility and DNA integrity are singled out in the acoustofluidic field. Reprinted with permission from [143]. (**D**) Schematic illustration of a standing surface acoustic wave (SSAW)-based microfluidic chamber to encourage sperm motility through acoustic actuation. Reprinted with permission from [145].

Beyond merely guiding object trajectories via tailored acoustofluidic fields, researchers have progressively unveiled that living cells, contingent upon their physiological states, demonstrate varying responses to acoustofluidic stimulation [137]. Referencing such insights, a range of acoustofluidic biophysical phenotyping platforms have been conceptualized. Notably, in 2018, the Greco team elucidated that surface acoustic wave (SAW) excitation (48.8 MHz) can engender considerable fluid recirculation within a Petri dish while imposing minimal thermal effects (as depicted in Figure 5A). This stimulation was correlated with a notable 36% increase in cell proliferation compared to a control [138]. The following year, the Devendran group observed that acoustofluidic exposure could attenuate cell adhesion, abate cell spread, and most markedly, augment cell metabolic activity without compromising cell viability [139]. Although the precise mechanisms remain to be delineated, these observations suggest that acoustofluidic activation can modulate biological activities in cells. In more recent advancements, Kim and colleagues embarked on preclinical explorations [140], discovering that an acoustically (96.7 MHz) actuated dynamic cell culture system could bolster the activity of natural killer cells, thereby enhancing their cytotoxicity against target tumor cells (as shown in Figure 5B). This revelation has been touted as a potential precursor to novel applications for functionally optimized immune cells.

Acoustofluidic phenotyping extends its utility from individual cell applications to encompass whole, living cells in motion. For example, Bhadra et al. introduced an acoustofluidic gym that utilized an on-chip polydimethylsiloxane (PDMS) chamber containing medium, which was placed centrally between a pair of interdigital transducers (IDTs). This setup facilitates a systematic examination of varying exercise protocols and their consequences on neuronal integrity. By adjusting the duration and power intensity of SAW actuation, an optimal exposure setting has been found that substantially mitigates neurodegenerations in both models. This research underscores the nuanced relationship between exercise parameters and neuroprotection in *C. elegans*, unveiling the acoustofluidic gym as an instrumental tool for further inquiries in this domain [141,142]. More clinically, Gai et al. have contributed an innovative, automated methodology for the selection of high-quality sperm, targeting the resolution of male infertility [143,144]. This method is anchored in the virtual Deterministic Lateral Displacement (vDLD) principle, which leverages surface acoustic waves (SAWs) (19.3 MHz) to selectively displace sperm cells within a microfluidic channel (Figure 5C). Sperm with standard morphological characteristics, heightened DNA integrity, and superior motility are segregated and transported across the channel, effectively distinguishing them from nonviable cells and detritus. This technique has demonstrated substantial enhancements in sperm quality, evidenced by over 50% improvement in vitality, more than 60% improvement in progressive motility, and an increase in DNA integrity exceeding 38%. Its automated functionality and the simultaneous assessment of multiple sperm quality parameters suggest its significant promise for advancing Assisted Reproductive Technologies (ART). Additionally, this research team has shown that high-frequency SAW (2 W, 19.28 MHz) application can elevate sperm motility (Figure 5D), presumably by intensifying the rate of intracellular metabolic processes and bolstering energy production [145]. Although the detailed molecular mechanisms underpinning acoustofluidic biophysical therapy remain to be fully elucidated, the observed efficacies of this approach are undeniable. Consequently, acoustofluidic mechanotherapy is regarded as having considerable potential to facilitate fertility treatments.

## 7. Acoustofluidic Droplet Printing

**Figure 6:**
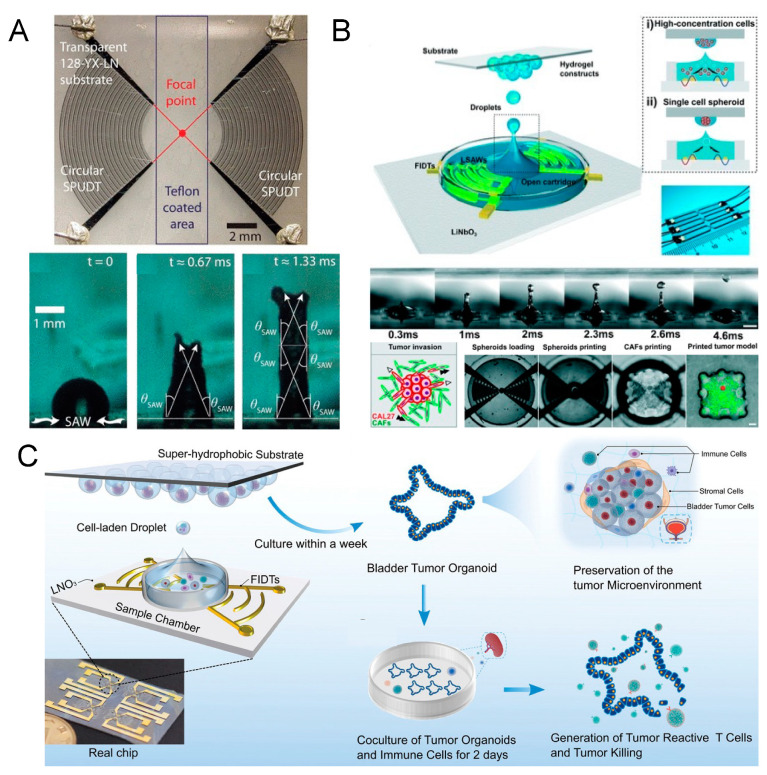
The acoustofluidic droplet printing systems for various biomedical applications. (**A**). Single-phase unidirectional transducers fabricated on lithium niobate substrate. The transducer pair is used to generate ultrasonic focus, inducing elongated liquid jets. Reprinted with permission from [151]. (**B**) Schematic drawing of acoustofluidic droplet printing. Both the single cells and single-cell spheroids can be located on demand. Reprinted with permission from [160]. (**C**) Schematic drawing of tumor organoid and the related microenvironment through the acoustofluidic droplet printing technique. Reprinted with permission from [161].

The surface acoustic wave-powered acoustofluidic droplet printing technique is an increasingly popular technique used to promote fluid–substrate interaction and realize three-dimensional spatial translocation of micro-droplets [146,147,148]. The most common concept is to use focused interdigital transducers (FIDTs) to transfer mechanical energy into a liquid and produce a jet to create tiny droplets [149,150]. Basically, two identical leaky surface acoustic waves (LSAWs) are released from opposing FIDTs and interfere with each other. Eventually, an intense acoustic pressure profile is formed at the geometrical focus to levitate the liquid–air interface above the acoustic energy’s focus [151]. When the mechanical vibration strength is strong enough to break the surface tension, the liquid above is deformed into elongated liquid columns (Figure 6A). Finally, tiny droplets are dispensed into the air [152]. Compared with the traditional water-in-oil droplet manufacturing method that uses the oil phase to shear the water phase, the acoustofluidic droplet printing technique gets rid of the limitations of the physical device channel and can directly create water-in-air droplets in an oil-free manner [153]. Without the conventional and complex oil–water separation operations, the droplets can be isolated and collected directly for subsequent applications. Further, the technique possesses the ability to precisely control the spatial position of a single droplet, unmatched by the resolution of traditional 3D bioprinting. Combined with a suitable hydrogel matrix, this technology is particularly suitable for constructing complex micro-physiological environments for in vitro tissue engineering research [154,155,156,157,158,159]. Chen et al. presented a functional tumor microenvironment constructed by the acoustofluidic droplet printing technique in 2021 (Figure 6B) [160]. They first demonstrated that a droplet array with a specific geometry can be generated on demand with the technique. Then, both the single-cell-laden droplet and single-cell spheroid-laden droplet printing processes were manifested. As a complete product display, the research group embedded a tumor spheroid right in the center of a single cancer-associated fibroblast (CAF)-rich microenvironment. They claimed that this advanced acoustofluidic bioprinting technique features high precision while maintaining low cell damage characteristics and is believed to support other in vitro disease model constructions. Soon after, Gong et al. reported tumor organoid formation through the same technique, proving that the technique can really deal with clinical samples (Figure 6C) [161]. Large-scale patient-derived bladder tumor organoids mimicking the in vivo immune microenvironment were produced within one week as part of an in vitro model to screen a potential personalized tumor immunotherapy. All these works testify that the technology can be used as a practical alternative to overcome the limitations of conventional 3D bioprinting technology when targeting microscopic models for in vitro investigation. 

## 8. Acoustofluidic Intracellular Delivery

**Figure 7:**
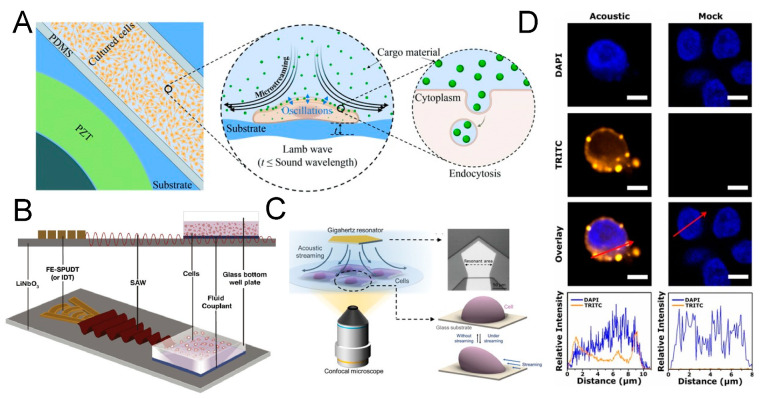
Acoustofluidic intracellular delivery into live cells. (**A**). An acoustofluidic device to generate acoustic streaming, enhancing the intracellular delivery of cargo into adherent cells. Reprinted with permission from [174]. (**B**). A SAW-based transfection platform to generate high-frequency acoustic excitation and enhance the uptake of RNA into nonadherent cells. Reprinted with permission from [175]. (**C**). A gigahertz ultrasonic device to induce controllable cells and, thus, adjust cellular membrane permeability. Reprinted with permission from [176]. (**D**). Confocal scanning results showing the fluorescence signal of delivered DNA material in cells with/without acoustofluidic sonoporation. The fluorescence intensity is profiled across the red arrows direction. Reprinted with permission from [177].

Intracellular cargo delivery is challenging but critical for many biotechnological applications, such as T-cell reprogramming for immunotherapy [162]. At present, electroporation is the most widely used strategy for transport across cell membranes but is still limited by throughput, efficiency, cost, and biocompatibility [163]. It is often difficult to control the electric field strength to favor the survival of most cells. Acoustofluidic delivery is based on mechanical effects and avoids the thermal and breakdown phenomena accompanying the electroporation effect; thus, it is expected to become a new-generation intracellular cargo delivery method that can be applied clinically [164,165,166,167,168]. The mechanism of acoustofluidic delivery is relatively complicated [169,170]. When acoustic waves propagate in a liquid containing cells, the cells are affected by both the acoustic radiation force and the shear force from the acoustic streaming flow. At the same time, tiny bubble nuclei in the liquid may be activated under the action of ultrasonic waves, manifesting as a series of dynamic processes such as the oscillation, growth, shrinkage, and collapse of the bubble nuclei, which is called ultrasonic cavitation. These bubbles may directly or indirectly act on cells to deform cell membranes, thereby affecting membrane permeability [171,172,173]. Salari et al. reported a relatively low-frequency (96 kHz) Lamb wave-based microfluidic BAW methodology for intracellular delivery in 2021 (Figure 7A) [174]. Once the acoustic source is activated, the adherent cells cultured nearby are subjected to mechanical oscillations from the underlying substrate and flow stress from the fluid above. The research team demonstrated the transport of cargo materials into the cells, including 500 kDa dextran, siRNA, and plasmids, without affecting cell viability. At the same time, Ramesan et al. reported a similar platform with high-frequency (30 MHz) acoustic excitation that could also adjust the membrane permeabilization for siRNA transport into nonadherent cells (Figure 7B) [175]. The same year, Guo et al. presented a high-resolution recording of a dynamic cell deformation process and membrane permeability modulation within their acoustofluidic delivery platform (1.64 GHz) (Figure 7C) [176]. It could be clearly observed that the adherent cells sway back and forth in the acoustic fluid field at ultra-high-frequency level, causing the deformation of the cell membrane. To examine whether intracellular delivery was achieved successfully, Belling et al. exploited confocal laser scanning to show that fluorescently labeled DNA were distributed throughout cell cytosol and on the cell membrane in acoustofluidically treated cells (Figure 7D) [177]. In addition to being applied to intracellular delivery at the single-cell level, researchers are also trying to combine this technology with wearable devices for controlled transdermal drug delivery at the tissue level, and the results are also promising [178,179]. Although it has natural advantages in preserving cell viability, the specific principles of this technology have yet to be confirmed qualitatively and quantitatively, which is fundamental for its future standardization and high-throughput scaling application. 

## 9. Summary and Outlook

The acoustofluidic manipulation of living cells represents an emerging and rapidly growing field with vast potential. Characterized by its contactless, non-altering, and benign bio-friendly nature, acoustofluidic techniques are particularly well suited for the manipulation of living cells [180,181]. With decades of laboratory research underpinning its development, this technology is becoming more widely adopted in laboratories worldwide, leading to a variety of innovative applications. Today, acoustofluidic technology can not only serve as a reliable assistant to facilitate fundamental biomedical research [182] but can also be integrated with automated robotic arm systems to create more advanced research instruments [183,184]. Moreover, the emergence of startups specializing in acoustofluidic technologies is a testament to its commercial viability and potential for growth. Looking ahead, there is an anticipation that acoustofluidics will converge with other technologies such as magnetic, optical, chemical, and artificial intelligence methods, thereby fostering new multidisciplinary technologies and therapeutic strategies [185,186,187,188,189,190,191,192]. Of course, there are still key bottlenecks in this technology that need to be solved. As a subdivision derived from microfluidic technology, most of the reported proof-of-concept devices were still fabricated with microfabrication techniques, such as photolithography and molding and replicas [193]. After plasma bonding piezoelectric wafers with PDMS material, researchers can customize acoustofluidic devices in a cleanroom in a fast manner. However, this silicone material in turn limits the large-scale manufacturing of proven devices. Plastic materials such as acrylic can be processed quickly, but there is no effective process to assemble them with piezoelectric materials without affecting acoustic field performance. In the future, the development of new materials or advances in processing techniques could further drive the true industrialization of acoustofluidic technology.

## Data Availability

No new data were created.
